# Induction of Robust B Cell Responses after Influenza mRNA Vaccination Is Accompanied by Circulating Hemagglutinin-Specific ICOS+ PD-1+ CXCR3+ T Follicular Helper Cells

**DOI:** 10.3389/fimmu.2017.01539

**Published:** 2017-11-13

**Authors:** Gustaf Lindgren, Sebastian Ols, Frank Liang, Elizabeth A. Thompson, Ang Lin, Fredrika Hellgren, Kapil Bahl, Shinu John, Olga Yuzhakov, Kimberly J. Hassett, Luis A. Brito, Hugh Salter, Giuseppe Ciaramella, Karin Loré

**Affiliations:** ^1^Department of Medicine Solna, Immunology and Allergy Unit, Karolinska Institutet, Stockholm, Sweden; ^2^Center for Molecular Medicine, Karolinska Institutet, Stockholm, Sweden; ^3^Valera LLC, Cambridge, MA, United States; ^4^Moderna Therapeutics, Cambridge, MA, United States; ^5^Department of Clinical Neuroscience, Karolinska Institutet, Stockholm, Sweden

**Keywords:** mRNA vaccine, adaptive immune responses, non-human primates, influenza, T follicular helper cells, germinal centers

## Abstract

Modified mRNA vaccines have developed into an effective and well-tolerated vaccine platform that offers scalable and precise antigen production. Nevertheless, the immunological events leading to strong antibody responses elicited by mRNA vaccines are largely unknown. In this study, we demonstrate that protective levels of antibodies to hemagglutinin were induced after two immunizations of modified non-replicating mRNA encoding influenza H10 encapsulated in lipid nanoparticles (LNP) in non-human primates. While both intradermal (ID) and intramuscular (IM) administration induced protective titers, ID delivery generated this response more rapidly. Circulating H10-specific memory B cells expanded after each immunization, along with a transient appearance of plasmablasts. The memory B cell pool waned over time but remained detectable throughout the 25-week study. Following prime immunization, H10-specific plasma cells were found in the bone marrow and persisted over time. Germinal centers were formed in vaccine-draining lymph nodes along with an increase in circulating H10-specific ICOS+ PD-1+ CXCR3+ T follicular helper cells, a population shown to correlate with high avidity antibody responses after seasonal influenza vaccination in humans. Collectively, this study demonstrates that mRNA/LNP vaccines potently induce an immunological repertoire associated with the generation of high magnitude and quality antibodies.

## Introduction

Emerging infections such as Ebola, Zika, Chikungunya, and pandemic influenza virus need vaccines that can be rapidly produced with antigen precision. Modified mRNA vaccines have received considerable attention as they are attractive in this aspect and were recently shown to induce sterilizing immunity to Zika virus infection and protection against lethal challenge with influenza virus (1–5). Advances in mRNA synthesis technology have led to increased mRNA stability, optimized translation capacity, and less indiscriminate activation of innate immunity by mRNA vaccines (2, 3, 6). mRNA vaccines do not require device-mediated delivery methods as with DNA vaccines, and there is no concern of pre-existing immunity typically associated with viral vector vaccine platforms (3). mRNA vaccines delivered in lipid nanoparticles (LNP) are well tolerated and highly immunogenic in mice, ferrets, non-human primates, and humans (5, 7). However, the fundamental mechanisms by which mRNA vaccines induce strong antibody responses are largely unknown.

Durable vaccine-induced antibody responses with a high degree of affinity maturation confer protection against most pathogens (8). Antibody-secreting plasma cells (PCs) in the bone marrow determine the magnitude and longevity of vaccine responses (8). PCs are derived from germinal centers (GCs) in secondary lymphoid tissues during the process of establishing humoral immunity after vaccination (9, 10). Within the dark zone of GCs, B cells that encounter cognate antigens undergo multiple rounds of proliferation and somatic hypermutation. This is followed by antibody affinity maturation in the GC-light zone in a T follicular helper (Tfh) cell-dependent manner (11). B cells, expressing high-affinity B cell receptors as a result of the GC reaction, differentiate into either memory B cells or PCs (12). Recently, a subset of CXCR5+ICOS+, programmed death receptor 1+ (PD-1) CD4+ T cells in the blood was termed circulating Tfh (cTfh) cells due to their similarities with Tfh cells in the lymph nodes (LNs) (13–15). As for regular T helper cells, cTfh cells can be subdivided based on cytokine profile and effector function (16). cTfh1 cells (CXCR3+) are proposed to excel at conferring protection against intracellular pathogens whereas cTfh2 cells (CXCR3− CCR6−) and cTfh17 (CXCR3− CCR6+) may be particularly important in the defense against extracellular pathogens and fungi (14). cTfh cells can be detected seven days after influenza vaccination in humans (17–19). CXCR3+ cTfh cells in humans as well as CXCR3+ Tfh cells in LNs of rhesus macaques have been reported to correlate with the generation of high-avidity antibodies following vaccination (17, 18, 20).

Details of the dynamics of B cell responses and the profile of T cell help induced by mRNA vaccines are currently not known. We, therefore, analyzed the development and maintenance of vaccine-specific responses to a non-replicating mRNA construct encoding the hemagglutinin (HA) of a pandemic influenza H10N8 strain (H10). We show that H10-specific CXCR3+ cTfh cells appeared in the blood 1 week after both prime and boost immunization. This was accompanied by robust GC formation in vaccine-draining LNs, a continuous increase in antibody avidity and seeding of H10-specific PCs to the bone marrow, altogether resulting in protective HA inhibition antibody titers sustained over the 25-week study period.

## Materials and Methods

### Production of Modified mRNA and LNP

Modified mRNA encoding the HA of H10N8 influenza A virus (A/Jiangxi-Donghu/346/2013) were generated as previously described (21). The lipid mixture was combined with a 50 mM citrate buffer (pH 4.0) containing mRNA at 3:1 ratio (aqueous:ethanol) using a microfluidic mixer (Precision Nanosystems). For formulations containing glucopyranosyl lipid adjuvant (GLA) (Avanti Lipids), lipids were combined in a molar ratio of 50:9.83:38.5:1.5:0.17 (ionizable lipid:DSPC:cholesterol:PEG-lipid:GLA). All formulations were dialyzed against PBS, concentrated using Amicon ultra centrifugal filters (EMD Millipore) and passed through a 0.22 µm filter. Particles were 80–100 nm in size with >95% RNA encapsulation.

### Immunizations and Sample Collection

This animal study was approved by the Local Ethical Committee on Animal Experiments. Chinese rhesus macaques were housed in the Astrid Fagraeus laboratory at Karolinska Institutet according to guidelines of the Association for Assessment and Accreditation of Laboratory Animal Care, and all procedures were performed abiding to the provisions and general guidelines of the Swedish Animal Welfare Agency. Animals were divided into three groups (*n* = 4/group) receiving H10 mRNA/LNP (50 µg) either by IM or ID delivery or H10 mRNA/LNP co-formulated with GLA adjuvant (5 µg) delivered IM. Prime and boost immunizations were delivered at week 0 and 4, respectively. Animals receiving H10 mRNA/LNP with GLA received an additional boost at week 15. The animals were sedated with ketamine 10–15 mg/kg given IM (Ketaminol 100 mg/ml, Intervet, Sweden) during the immunizations, blood and bone marrow sampling. Bone marrow was sampled before immunization and at 2, 6, and 25 weeks from the humerus as previously described (22).

An axillary LN was collected before vaccination, opposite from the planned vaccination site, and a collateral axillary LN after boost. The animals were anesthetized by IM injection of 10–15 mg/kg of ketamine and 0.05 mg/kg of medetomidine. Carprofen (4 mg/kg) was given IM as analgesia. LNs were removed in an aseptic manner using minimal entry holes with the aim of removing a singular LN in each procedure. The anesthesia was reversed with atipamezole, 0.25 mg/kg IM after suturing.

### Blood and Bone Marrow Processing

Peripheral blood was drawn into EDTA tubes and peripheral blood mononuclear cells (PBMCs) were isolated using Ficoll-Paque™ PLUS (GE Healthcare) and washed with PBS. Red blood cells were removed using red blood cell lysis buffer and cells were frozen in 10% DMSO (Sigma-Aldrich) diluted in heat-inactivated FBS. Bone marrow mononuclear cells were isolated and stored in a similar manner as PBMCs.

### Lymph Node Processing

Lymph nodes were processed as previously described (23). Briefly, LNs were cleaned of fat and cut into small pieces using surgical scissors before being minced and filtered through 70 µm cell strainers. The cells were frozen as described above.

### Hemagglutination Inhibition (HAI) Assay

Hemagglutination inhibition assay was performed using 0.5% turkey red blood cells (Rockland Antibodies and Assays) diluted in PBS to investigate protective antibody titers. Serum was incubated overnight at +37°C with receptor destroying enzymes (Denka Seiken) to prevent non-specific HAI. Serial dilutions (1:2) of serum samples were performed in V-bottom 96-well plates in duplicates, starting from 1:10 dilution. Recombinant HA of H10N8 influenza A virus (4 units), A/Jiangxi-Donghu/346/2013 (Medigen Inc.) were added to diluted serum and incubated for 30 min at room temperature. The reciprocal of the last serum dilution that resulted non-agglutinated red blood cells represented the HAI titer. Titers <10 were assigned as 1.

### IgG Avidity ELISA

Clear flat-bottom immune 96-well plates (Thermofisher) were coated for 3 h in 37°C with 100 ng of H10 protein per well. After washing, wells were blocked for 1 h at 37°C in 2% milk powder (blocking buffer) diluted in PBS. Blocking buffer was discarded and plasma samples diluted in blocking buffer were added and incubated for 1 h at 37°C. Plasma was discarded and wells were incubated for 10 min with PBS or sodium thyocyanate (NaSCN) at 1.5–4.5M. After washing, wells were incubated with anti-monkey IgG HRP antibody (Nordic Labs) in washing buffer (0.05% Tween20 in PBS) for 1 h at 37°C. After washing, 100 μl of TMB solution (BioLegend) was added to each well, the reaction was stopped at 5 min with 100 μl of 1M sulfuric acid. Wells were read at 450 nm using an ELISA reader (PerkinElmer).

### B Cell ELISpot

The frequency of H10-specific antibody-secreting cells (ASCs) and memory B cells was determined as previously described (24), with some modifications. In brief, MAIPSWU10 96-well plates (Millipore) were coated with 10 µg/ml of anti-human IgG (Fcγ; Jackson ImmunoResearch Laboratories). Cells were transferred in duplicate dilution series and cultured overnight at 37°C. For enumeration of ASCs, cells were plated directly without prior stimulation, whereas for memory B cells, cells were prestimulated for 4 days at 2 × 10^6^ cells/ml with 5 µg/ml CpG-B (ODN 2006; Invivogen), 10 µg/ml Pokeweed mitogen (PWM; Sigma-Aldrich), and 1:10,000 Protein A from Staphylococcus aureus Cowan strain (SAC; Sigma-Aldrich). Plates were washed with PBS containing 0.05% Tween-20 (PBS-T), then incubated with 0.25 µg/ml biotinylated goat anti-human IgG (Fcγ; Jackson ImmunoResearch Laboratories) for total IgG determination, 0.1 µg/ml biotinylated H10 for H10-specific determination, or 0.1 µg/ml biotinylated OVA in PBS-T. The plates were washed and then incubated with streptavidin-conjugated alkaline phosphatase (Mabtech) diluted 1:1,000 in PBS-T. Spots were developed with BCIP/NBT substrate (Mabtech) and counted using an AID ELISpot Reader and version 6 of the accompanying software (Autoimmun Diagnostika). Unspecific spots were subtracted from antigen-specific wells, as defined by spots counted in samples incubated in OVA-probed wells.

Recombinant HA of H10N8 influenza A virus (H10), A/Jiangxi-Donghu/346/2013 (Medigen Inc.), and ovalbumin (OVA; Invivogen) molecules were biotinylated using the EZ-Link Sulfo-NHS-Biotinylation kit (Thermo Fisher) using a 1:1 M ratio and unreacted biotin was removed using the included Zeba Spin Desalting Columns.

### Phenotypic Analysis and H10 Recall Assay of Tfh Cells

1.5 × 10^6^ cells from indicated time points were stained with LIVE/DEAD Fixable Blue Dead Cell kit according to manufacturer’s protocol (Invitrogen). Samples were surfaced stained with a panel of fluorescently labeled antibodies (Table S1 in Supplementary Material) to identify specific cell subsets. Additionally, 1.5 × 10^6^ PBMCs were rested for 3 h and then stimulated overnight in complete media (10% FCS, 1% penicillin/streptomycin/glutamine in RPMI, all from Gibco, Stockholm, Sweden) in *U*-bottom 96-well plates with H10 peptides (15mers overlapping by 11 amino acids, 2 µg/ml) and Brefeldin A at 10 µg/ml. Cells were stained with surface-specific antibodies (Table S1 in Supplementary Material), fixed, and permeabilized using fixation and permeabilization solution (BD Biosciences) before being stained for intracellular cytokines (Table S1 in Supplementary Material). Samples were resuspended in 1% paraformaldehyde before acquisition using a Fortessa flow cytometer (BD Biosciences). Results were analyzed using FlowJo version 9.7.6. Background cytokine staining was subtracted, as defined by staining in samples incubated without peptide.

### Detection of GCs *In Situ*

Extirpated LNs were snap frozen using dry ice in OCT media (Tissue-Tek) and kept in −80°C until use. Tissues were thawed to −20°C and then sectioned (8 µm) and fixed for 15 min in 2% formaldehyde in PBS. Tissues were permeabilized using tris-buffered saline with 0.1% saponin and 1% hepes buffer (permwash with pH 7.4), all future reagents were diluted in permwash. LNs were blocked with 1% FCS and then stained with anti CD3 (Dako), Ki67 (BD), and PD-1 (R&D systems). After this, biotinylated anti-mouse or anti-goat (Dako) or anti-rabbit (Vector Labs) secondary antibodies were added, which were detected by the addition of streptavidin-conjugated Alexa Fluor 405/555/647 (Invitrogen). Image tiles of entire LNs were acquired using a Nikon Eclipse Ti-E confocal microscope. GCs were defined as dense follicular structures including CD3+PD-1+ cells (light zone) and Ki67+ cells (dark zone) (Figure 3A). Image analysis was done using CellProfiler software (Broad Institute Inc.) with in-house algorithms. Briefly, GCs were manually identified in the program to enable automatic enumeration of PD-1+ and Ki67+ cells within the individual GCs and the area of the GCs. PD-1+ cells were almost exclusively CD3+ and Ki67+ cells were mostly CD3− (GC B cells).

### CXCL13 ELISA

Undiluted plasma stored in −20°C was thawed and analyzed using a Quantikine Human CXCL13/BLC/BCA-1 Elisa (R&D systems) according to manufacturer’s instructions.

### Detection of Antigen-Specific GC B Cells by Flow Cytometry

2 × 10^6^ cells from LN cell suspensions were stained with LIVE/DEAD Fixable Blue Dead Cell kit according to manufacturer’s protocol (Invitrogen). Samples were then incubated with a titrated amount of H10-tetramer probe for 20 min at 4°C. H10-tetramer probes were prepared beforehand by mixing biotinylated H10 protein with streptavidin-BV421 (Biolegend) at a 4:1 M ratio. Samples were washed and stained with a surface antibody cocktail (Table S2 in Supplementary Material). Before intracellular staining (Table S2 in Supplementary Material), samples were fixed and permeabilized using the Transcription Factor Buffer Set (BD Biosciences). Samples were resuspended in 1% paraformaldehyde before acquisition using a Fortessa flow cytometer (BD Biosciences). Results were analyzed using FlowJo version 9.7.6.

### Statistical Analysis

Statistical analysis was conducted by Prism Version 6.0 (GraphPad) software. All of the data are presented as the mean ± SEM. Difference between groups was analyzed as described in figure legends. A *p*-Value <0.05 was considered to be statistically significant.

## Results

### mRNA Vaccine Encoding H10 Induces Protective Levels of Antibodies

**Figure 1:**
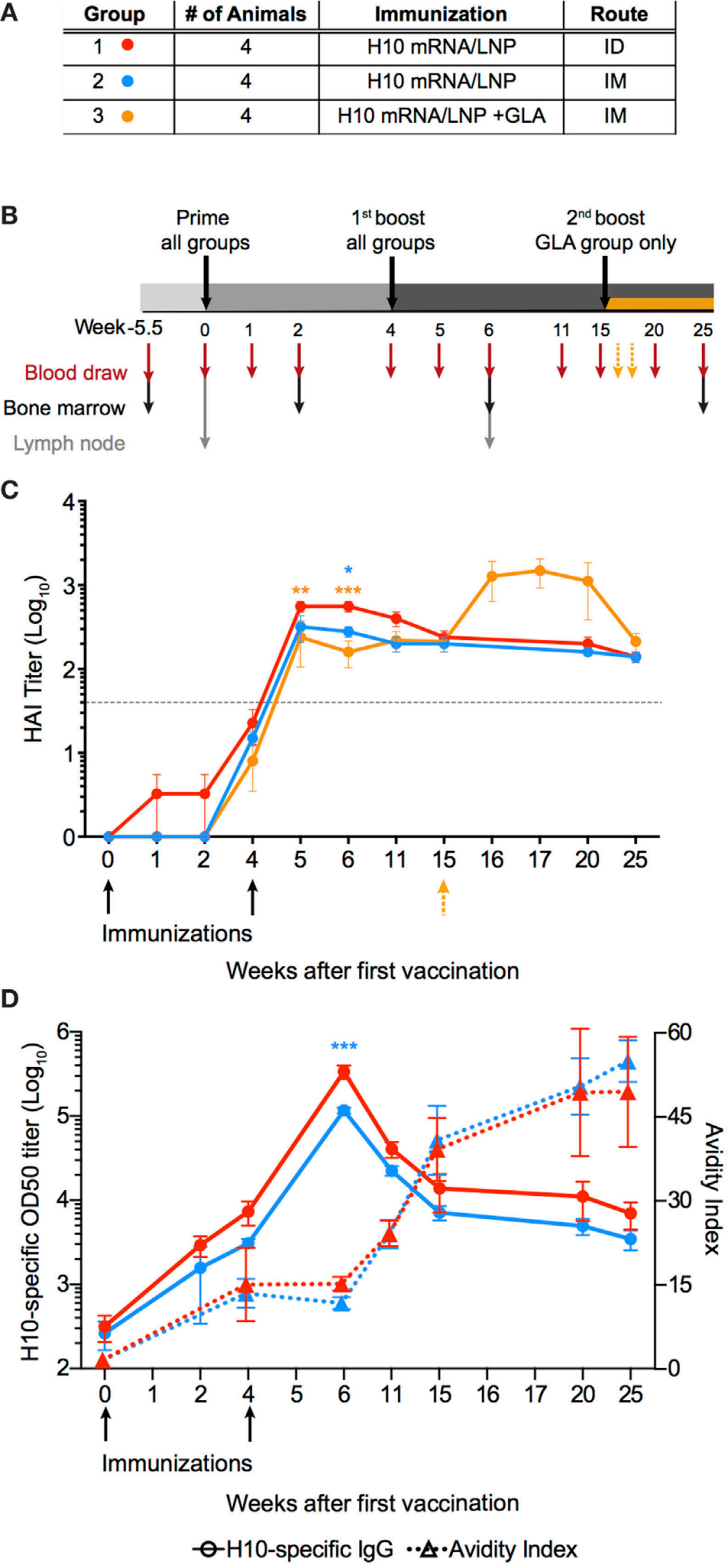
mRNA vaccine encoding influenza H10 elicits high antibody titers by intradermal and intramuscular immunization. Rhesus macaques were vaccinated with mRNA encoding for hemagglutinin of H10N8 influenza encapsulated in LNPs. **(A)** Shows the study groups and **(B)** shows the immunization and sampling schedule. **(C)** Antibody levels over time induced in the different groups as assayed by hemagglutination inhibition assay. Arrows indicate immunizations at week 0 and 4, as well as 15 for the glucopyranosyl lipid adjuvant group only. **(D)** The left *y*-axis shows antigen-specific plasma IgG titers (circles) and the right *y*-axis shows avidity index (triangles). Arrows indicate immunizations at week 0, 4. Titers are displayed as mean ± SEM. Dotted line depicts the accepted level of protection for seasonal influenza. Statistics was calculated by two-way ANOVA with Tukey’s multiple comparison test: *p < 0.05, **p < 0.01, ***p < 0.001.

Rhesus macaques were immunized either intramuscularly (IM) or intradermally (ID) with an mRNA vaccine encoding the full-length HA of H10N8 (A/Jiangxi-Donghu/346/2013) (H10) formulated in LNP (Figure 1A). A third group received this formulation combined with the TLR4-agonist GLA to evaluate whether an adjuvant could further enhance immune responses. All animals received a homologous prime-boost immunization at 0 and 4 weeks (Figure 1B). In addition, the GLA group received a third immunization at 15 weeks.

All animals induced neutralizing antibody titers against HA above the accepted level of protection for seasonal influenza vaccination, as measured by hemagglutination inhibition assay (HAI) (Figure 1C) (25). Although some of the animals in the ID group already showed titers at the protective level after the prime immunization, all groups had titers that exceeded this level following boost. The antibody levels persisted above this level for the remainder of the study. The titers were significantly higher in the ID group compared to the IM groups for up to 2 weeks following boost, but were similar thereafter. The GLA group did not show higher HAI titers compared to the other groups, thus indicating that the mRNA/LNP formulation itself was sufficiently immunogenic. The third immunization in the GLA group resulted in a transient increase in HAI titers, which returned to similar levels as the other groups 5 weeks later.

Antibody titer, as measured by HAI, is a function of both the quantity and quality of an antibody. However, by combining these two measurements, certain mechanistic insights into antibody development may be lost over time. Therefore, total IgG titers against H10 as well as antibody avidity were measured separately. H10-specific IgG antibody titers were induced in both IM and ID groups after prime immunization. Titers continued to increase at the time of the second immunization and peaked 2 weeks thereafter (Figure 1D). The avidity index of H10-specific IgG antibodies as measured by a chaotropic ELISA wash assay, did not increase between 4 and 6 weeks following the second immunization (Figure 1D). However, at week 11, there was a clear increase in avidity, which continued to rise until the study end. There were no significant differences in IgG titers or avidity between the ID and IM group, except in the ID group two weeks after boost, which had higher IgG titers (*p* < 0.0001). Collectively, this demonstrates that two immunizations with the H10 mRNA/LNP formulation were sufficient to induce protective and durable antibody titers.

### Rapid and Sustained B Cell Responses after mRNA Vaccination

**Figure 2:**
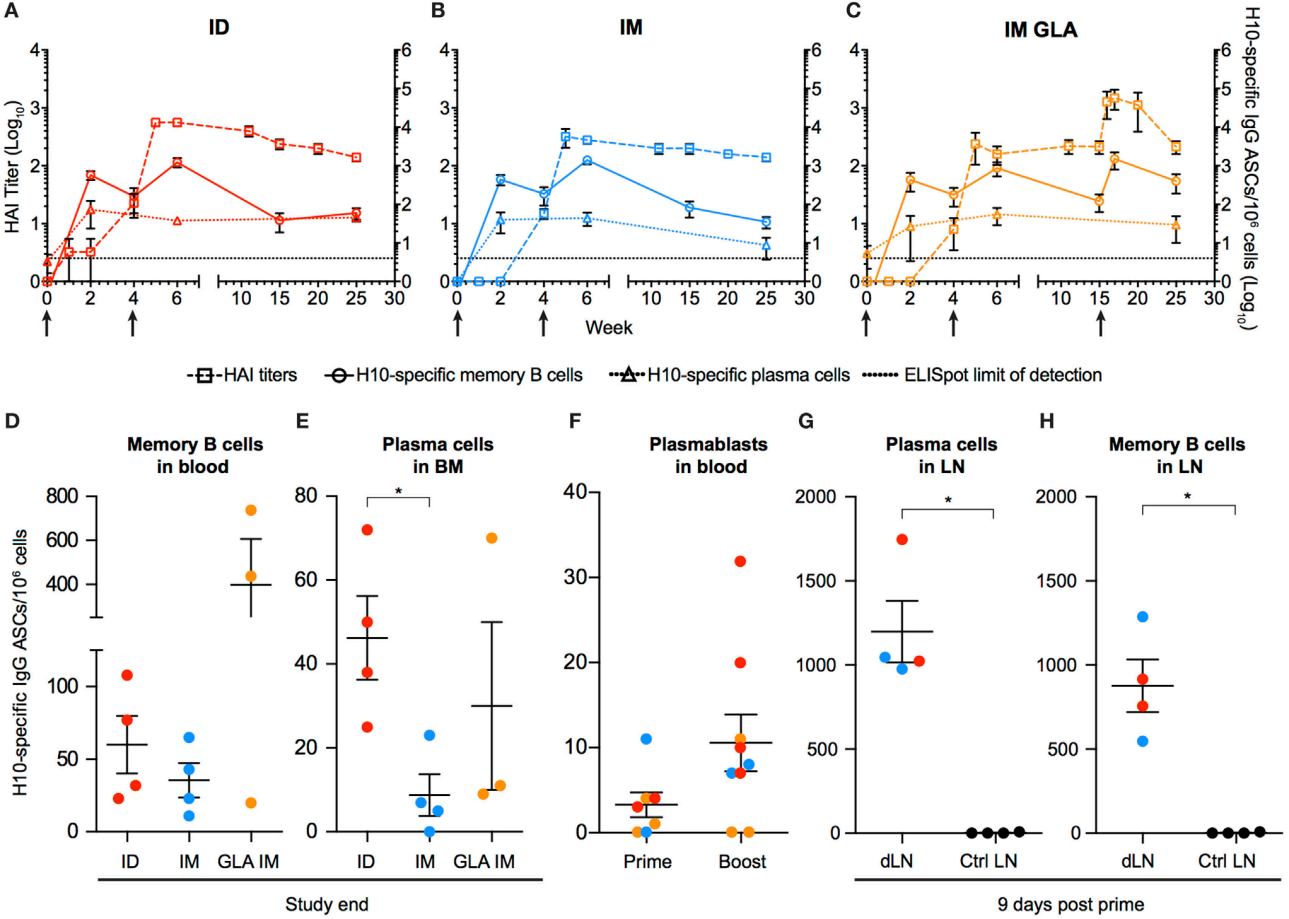
Kinetics of vaccine-induced antibody-secreting cells (ASCs) in bone marrow and blood. **(A–C)** The left *y-*axis shows HAI titers (squares). The right *y*-axis shows H10-specific cultured memory B cells in blood (circles) and H10-specific plasma cells (PCs) in bone marrow (triangles) determined as ASCs per 1 × 10^6^ cells. ASCs were plotted with HAI (squares) for comparison. Dotted line depicts the limit of detection for the ELISpot assay. **(D–E)** Study end values of antigen-specific memory B cells **(D)** and bone marrow PCs **(E)** between the groups. **(F)** Antigen-specific plasmablasts per 1 × 10^6^ cells 7 days after prime and boost. **(G,H)** Naïve animals were immunized and LNs were collected after 9 days. Antigen-specific PCs **(G)** and memory B cells **(H)** in vaccine-draining LNs vs non-draining control LNs. Red colored dots corresponding to intradermal immunization, blue to IM immunization, and orange to IM immunization with glucopyranosyl lipid adjuvant adjuvant. Data show mean ± SEM. Statistics was based on Mann–Whitney *U*-test *p < 0.05.

To characterize the kinetics of the B cell responses at the cellular level, the frequency of H10-specific memory B cells was determined by ELISpot (Figures 2A–C). Circulating H10-specific memory B cells were readily detectable 2 weeks after the prime immunization (Figures 2A–C). Thereafter, the number of H10-specific memory B cells contracted slightly, but expanded following the boost immunization. This was followed by a gradual decline. The GLA group showed an additional increase 2 weeks after the second boost as expected (Figure 2C). By study end (25 weeks), the IM and ID groups showed similar levels of circulating H10-specific memory B cells (Figure 2D), whereas the GLA group had higher levels due to the second boost.

Similar to the rapid induction of circulating H10-specific memory B cells, we detected H10-specific PCs in the bone marrow 2 weeks after the prime immunization in all groups tested (Figures 2A–C). However, in contrast to the memory B cell pool, the number of PCs remained relatively stable throughout the study. This dichotomy in fluctuation between memory B cells and PCs is in line with previous reports on protein vaccine immunizations (24).

The number of H10-specific PCs declined significantly in the IM group compared to the ID group by the end of the study (Figure 2E). Next, we analyzed plasmablasts in some of the animals at one week after immunization. The numbers of H10-specific plasmablasts were close to the limit of detection after the prime immunization, but increased to readily detectable levels after the boost immunization (Figure 2F).

We had previously shown that the priming of vaccine-specific T cells occurs in the vaccine-draining LNs (23). To investigate whether the priming of B cells also takes place at this site, vaccine-draining LNs were collected 9 days after prime immunization and compared with control LNs that did not drain the vaccine delivery site. H10-specific memory B cells and PCs were detected only in the vaccine-draining LNs (Figures 2G,H). This was observed with both ID and IM administration. This demonstrates that the H10 mRNA/LNP vaccine induces robust levels of vaccine-specific memory B cells and PCs following prime immunization and that these levels are sustained.

### GC Formation in Vaccine-Draining LNs

**Figure 3:**
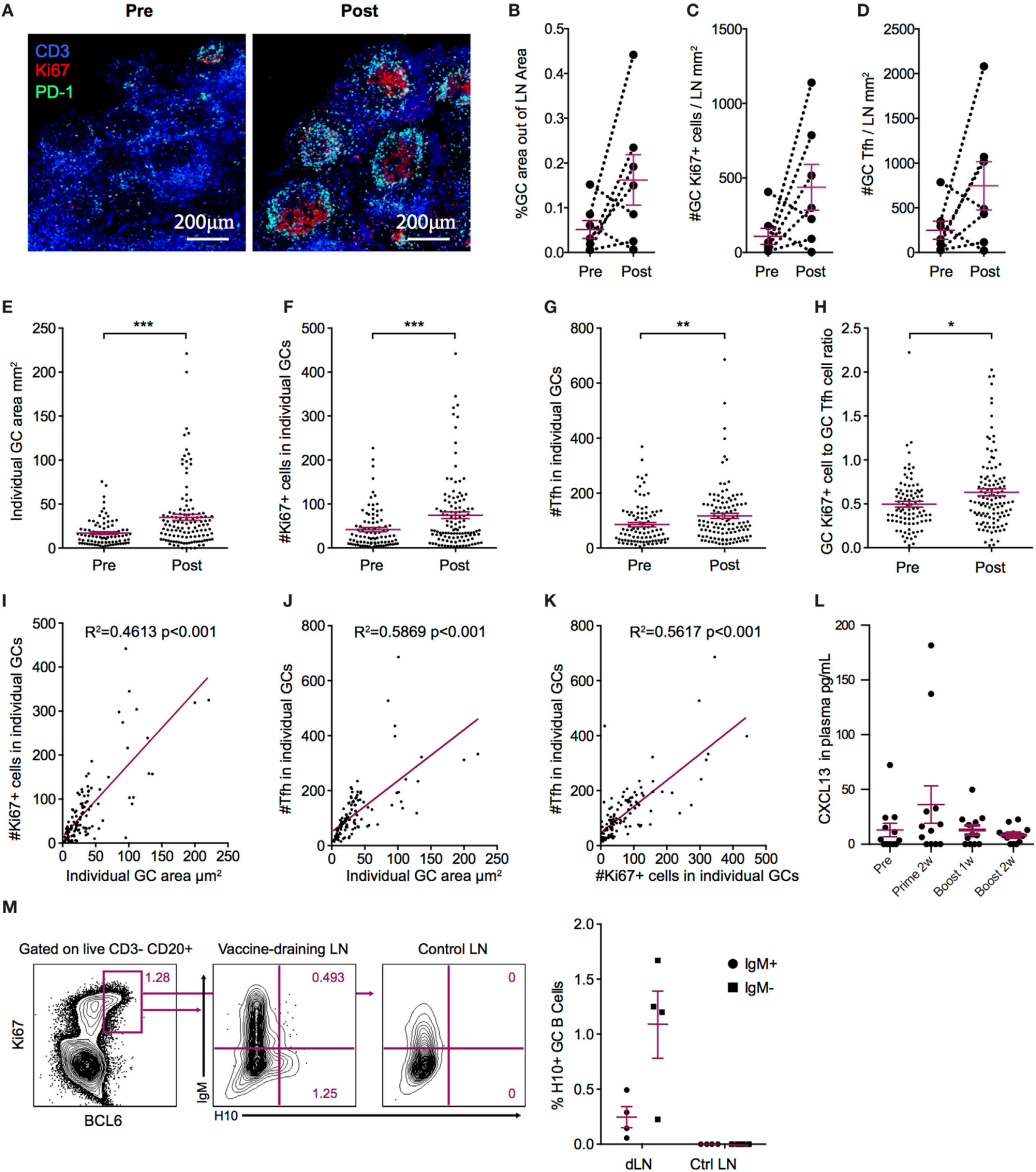
Increased germinal center (GC) activation post influenza mRNA vaccine immunization. Axillary lymph nodes (LNs) were obtained before prime and 2 weeks after boost. A variety of readouts for GC activity were evaluated following the boost immunization. **(A)** LN sections stained for GCs before and after vaccination. Sections were stained with anti-CD3 (blue), PD-1 (green), and Ki67 (red) and follicular structures with CD3+PD-1+ T follicular helper (Tfh) and Ki67+ cells were considered GCs. Images were analyzed with CellProfiler software. **(B–D)** Each LN was analyzed for average GC area and total number of Ki67+ cells and Tfh cells, divided by the LN area for normalization. **(E–H)** Individual GCs represented by dots. **(E)** GC area. **(F)** Number of Tfh cells. **(G)** Number of Ki67+ cells. **(H)** Ki67+ to Tfh cell ratio. **(I–K)** GC architecture homogeneity was investigated by plotting GC area, Tfh, and Ki67+ cell numbers against each other. **(L)** CXCL13 in plasma at indicated time points. **(M)** Antigen-specific GC (CD3− CD20+ BCL6+ Ki67+) B cells in vaccine-draining LNs 9 days after prime vs non-draining control LNs by flow cytometry using an H10 tetramer probe. Data show mean ± SEM. Nonparametric Wilcoxon matched-pairs signed rank test was used for **(B–D)** and Mann–Whitney *U*-test for **(E–H)**. *p < 0.05, **p < 0.01, ***p < 0.001.

Durable levels of H10-specific memory B cells and PCs coupled with a steady increase of IgG antibody avidity induced by the H10 mRNA/LNP vaccine are likely the outcome of a strong GC reaction. Since priming of H10-specific B cells appeared to be restricted to the vaccine-draining LNs, we also analyzed the expansion of GCs at this site. Using immunofluorescence and confocal imaging of cryosections, we quantified the area of GCs, the numbers of PD-1+ Tfh cells, and proliferating Ki67+ cells within individual GCs (26) (Figure 3A). Since the HAI titers did not differ between the three study groups, we did not stratify the data by group for this analysis. We evaluated LNs where biopsies from the same animal could be obtained both before immunization and 2 weeks post-boost. All vaccine groups were represented among the seven animals where such set of LNs was available (*n* = 1 from group 1, *n* = 4 from group 2, and *n* = 2 from group 3). We normalized the total GC area and total numbers of GC Tfh cells or GC Ki67+ cells by LN area. In five out of the seven animals, there was an increase in the GC area/LN area ratio post-immunization (Figure 3B). There was also an increase in GC Ki67+ cells/LN area ratio and GC Tfh cells/LN area ratio (Figures 3C,D). One advantage of analyzing tissue sections over cell suspensions is the option to study individual GCs in intact LN architecture rather than all GC cells combined. The area of individual GCs was significantly increased post-vaccination (Figure 3E). This was also observed for Tfh cells and GC Ki67+ cells within individual GCs (Figures 3F,G). As expected, the increase in the number of GC Ki67+ cells was greater than the increase in the number of GC Tfh cells, since GC B cells have an enhanced capacity for proliferation (9). To this end, it has been shown that the B cell/Tfh cell ratio is higher in the top neutralizers following HIV Env vaccination (27). We observed a significant increase in the Ki67+ cell/Tfh cell ratio post-vaccination in individual GCs (Figure 3H). There was a strong positive correlation between the number of Ki67+ cells and the area within each GC (*p* < 0.001, *R*^2^ = 0.4613) (Figure 3I). This was also found for the number of GC Tfh cells and the GC area of individual GCs (*p* < 0.001, *R*^2^ = 0.5869) (Figure 3J). Additionally, Tfh cell numbers correlated with Ki67+ cell numbers within each GC (*p* < 0.001, *R*^2^ = 0.5617) (Figure 3K).

Increased levels of the chemokine CXCL13 in plasma was recently proposed as a biomarker for GC activity in the LNs (28). There was a modest elevation in CXCL13 levels detected transiently 1 week after prime vaccination, with no noticeable increase observed at 1 or 2 weeks following boost vaccination (Figure 3L). GC formation after prime vaccination was also investigated using flow cytometry by enumerating the frequency of BCL6+ Ki67+ GC B cells expressing H10-specific B cell receptors in the vaccine-draining LNs (Figure 3M). No H10-specific GC B cells were found in control non-draining LNs, but both unswitched IgM+ and class-switched IgM− BCL6+ Ki67+ GC H10-specific B cells were detected in vaccine-draining LNs 9 days after prime immunization (Figure 3M). Taken together, this suggests that the mRNA/LNP vaccine is a potent inducer of GC activity.

### Circulating H10-Specific ICOS+ PD-1+ CXCR3+ Tfh Cells Are Induced after Vaccination and Correlate with Antibody Avidity

**Figure 4:**
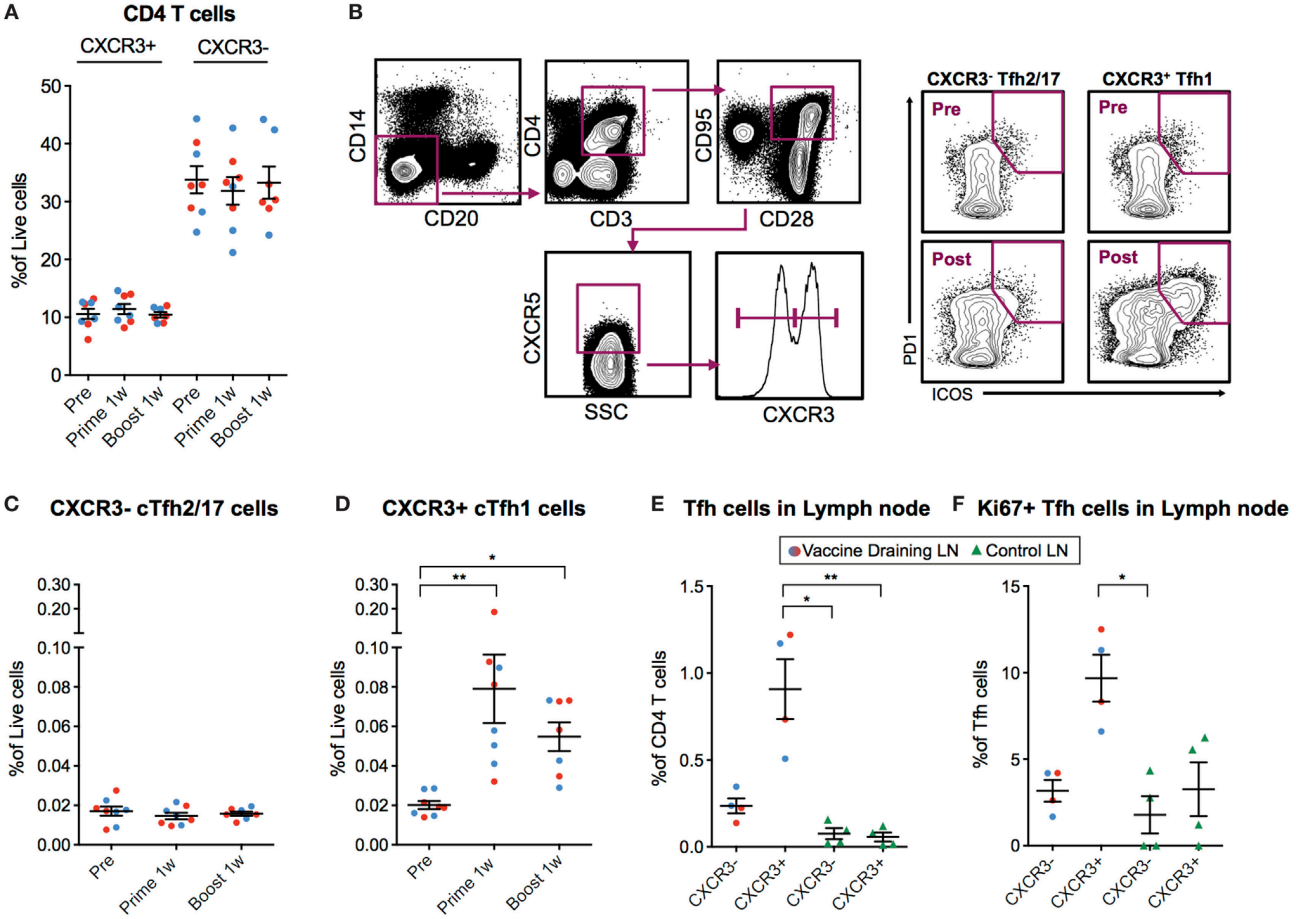
Circulating CXCR3+ T follicular helper (cTfh) cells are detected 7 days postvaccination. **(A)** Peripheral blood mononuclear cells (PBMCs) from before vaccination and 1 week after prime and boost immunization were phenotyped for the frequency of CXCR3 +/− CD4 T cells. cTfh cells from the same time points were enumerated using the gating strategy as indicated in **(B)**. Lymph node cell suspensions after prime immunizations and non-draining LNs were phenotyped for the frequency of Tfh cells and Ki67+ cells using the gating strategy as indicated in **(B)**. **(C,D)** Show the percentage of CXCR3− vs CXCR3+ cTfh cells out of live cells, respectively. **(E)** CXCR3+/− Tfh cells out of CD4+ T Cells in vaccine- and non-draining lymph nodes (LNs). **(F)** Ki67+ cells out of CXCR3+/− Tfh cells in vaccine- and non-draining LNs. Data from the ID and IM group are shown as red and blue, respectively. Data show mean ± SEM. Statistics was based on Wilcoxon matched-pairs signed rank test for **(C,D)** and Kruskal–Wallis test with Dunn’s multiple comparison for **(E,F)**, *p < 0.05, **p < 0.01.

As mentioned above, cTfh cells can be identified in blood as CXCR5+ICOS+PD-1+ CD4+ T cells. The Th1-polarized CXCR3+ cTfh cell subset specifically has been shown to correlate with high-avidity antibodies 7 days after influenza vaccination in humans (17, 18). Therefore, we investigated cTfh cell subset frequencies pre-vaccination and 7 days following prime and boost. We first concluded that there was no general increase in CXCR3+ or CXCR3− total CD4+ T cells (Figure 4A). Next, we analyzed CXCR5+ ICOS+ PD-1+ CXCR3+/− cTfh cells within the central memory (CD28+CD95+) CD4+ T cell population (Figure 4B). While there was no increase in CXCR3− cTfh cells (Figure 4C), there was a significant increase in the number of CXCR3+ cTfh cells both after prime and boost (Figure 4D). No notable differences in overall cTfh cells were found between the ID and IM groups.

We investigated whether the CXCR3+ cTfh cells were expanded in the vaccine-draining LNs before entering the circulation by analyzing the set of LNs collected at nine days post-prime. The frequency of Tfh cells was increased in the vaccine-draining LNs compared to control non-draining LNs (Figure 4E). In addition, the majority of the Tfh cells in the vaccine-draining LNs expressed CXCR3 (Figure 4E). CXCR3+ Tfh cells in vaccine-draining LNs also expressed Ki67 at higher levels than CXCR3− Tfh cells in the same and CXCR3+/− Tfh cells from control LNs (Figure 4F). Although difficult to prove, these findings indicate that the cTfh1 polarized cells found in peripheral blood after mRNA vaccine administration may have been generated in the vaccine-draining LNs.

**Figure 5:**
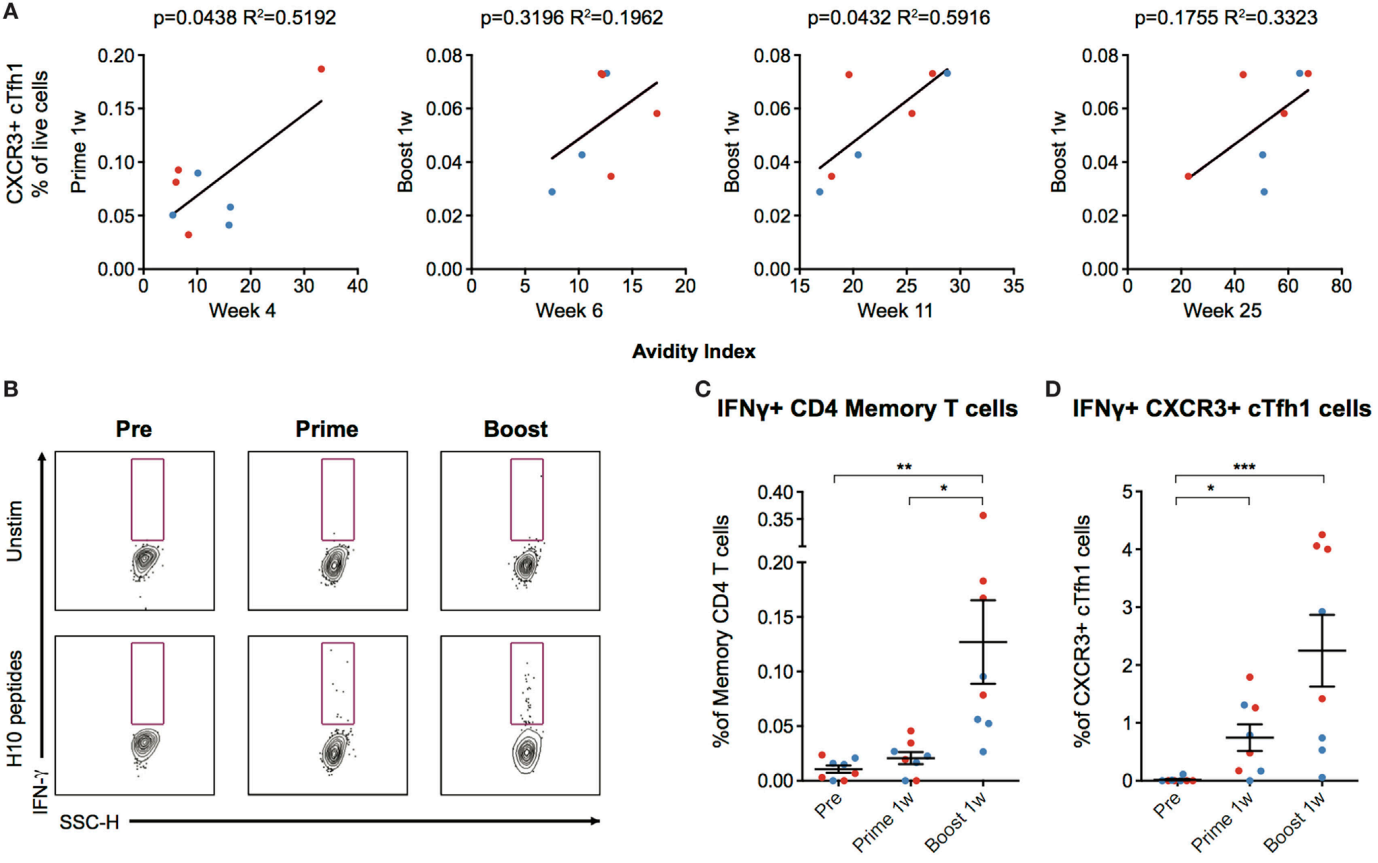
Circulating CXCR3+ T follicular helper (cTfh) cells correlate with antibody avidity and are antigen-specific. **(A)** Correlations between circulating CXCR3+ cTfh cells after prime and boost and avidity index at indicated time points. **(B)** Peripheral blood mononuclear cells were stimulated overnight with an H10 overlapping peptide pool (15mers overlapping by 11 amino acids) to recall H10 specific T follicular helper (Tfh) cells within the memory T cell pool. Representative gates showing IFNγ+ cells within CXCR3+ Tfh cells as gated in Figure 4B. **(C)** Percentage of IFNγ+ cells within the overall CD4 T cell memory pool after background subtraction. **(D)** Percentage of IFNγ+ cells within the CXCR3+ cTfh cells after background subtraction. Data from the ID and IM group are shown as red and blue, respectively. Data show mean ± SEM. Statistics were based on Friedman’s test with Dunn’s multiple comparison *p < 0.05, **p < 0.01, ***p < 0.001.

The number of CXCR3+ cTfh cells following boost correlated with antibody avidity at week 6 and 25, with a significant correlation at week 11 (*p* = 0.0432, *R*^2^ = 0.5916) (Figure 5A). To investigate cTfh cell specificity, PBMCs were stimulated with H10 overlapping peptides and tested for cytokine secretion by a standard intracellular cytokine assay (Figure 5B). There were few H10-specific cells as evidenced by IFNγ production within the total CD4+ central memory T cell population 1 week post-prime, but there was a clear increase one week following boost (Figure 5C). The peptide stimulation and culture procedure resulted in some loss in the resolution of CXCR5+ICOS+PD-1+CXCR3+ cTfh cellular staining. However, a significant increase in the number of IFNγ+CXCR3+ cTfh cells 1 week after prime and boost was still observed (Figure 5D). Collectively, this demonstrates that the H10/LNP vaccine induces H10-specific cTfh cells of the CXCR3+ Th1-polarized profile that correlate with the avidity of H10-specific IgG antibodies.

## Discussion

mRNA vaccines can be produced rapidly with high antigen precision, which offers an advantage over the more time-consuming process of producing live-attenuated or subunit protein vaccines (3, 29). Due to antigenic shifts and drifts, the influenza vaccine is renewed yearly to protect against the circulating strains. The mRNA platform would, therefore, be suitable for seasonal influenza vaccines as well as pandemic influenza strains (4, 5, 29). Although mRNA vaccines have already advanced to clinical trials, little is known of the adaptive immune profile induced by such vaccines. Therefore, this study focused on key immunological events resulting in antibody responses to an mRNA/LNP vaccine, using rhesus macaques whose immune system closely resembles that of humans. Influenza HA was chosen as the antigen due to the clinical relevance for the mRNA platform and that the levels of antibodies required for protection against seasonal influenza are well-characterized and could be benchmarked against (25). However, important to note is that all the animals in our study were naïve to influenza before immunization, which is in contrast to a clinical setting where most humans will have some degree of pre-existing immunity due to vaccination or prior infection. Therefore, the presence of cross-reactive HA-specific memory B cells and T cells may result in a stronger priming response than recorded in the rhesus macaques, although pre-existing immunity to HA of the pandemic influenza strain H10N8 used in this study is relatively rare in humans.

Seasonal influenza vaccines have been shown to induce protection in 60–90% of recipients (30, 31). In our study, we found that two animals in the ID group reached the accepted level of protection as measured by HAI titers after prime immunization. Animals across the groups reached this level after boost immunization and remained above this level for the entire 25-week study. It is plausible that all animals would have reached protective levels after the prime immunization if more time had been allowed since both the HAI and IgG titers were gradually increasing prior to the boost.

The memory B cell pool expanded quickly after prime immunization, suggesting that the mRNA platform is efficient at priming naïve B cells. H10-specific IgG titers were readily detectable 2 weeks after prime, but neutralization capacity, as measured by HAI titers, was found in a single animal at this time point. This could be explained by the initial low affinity of the memory B cell population, which would lead to insufficient neutralization.

The seeding of PCs into the bone marrow was also detected at 2 weeks post-prime immunization in all groups. The frequency of vaccine-specific PCs that were generated with the mRNA vaccine is comparable to that reported with recombinant HA protein co-administered with a strong adjuvant in the same animal model (24). Also, additional boosts did not increase the PC compartment in the bone marrow with either of the vaccine platforms (24). Regulation of the PC pool in the bone marrow is not well known. We found that the PC levels remained relatively constant once established after the prime, despite HAI titers and H10-specific IgG titers increasing significantly post-boost. H10-specific IgG titers waned more rapidly than HAI titers. This may be explained by the observed increase in antibody avidity, which suggests increased survival of high affinity PCs and/or ongoing affinity maturation in GCs long after immunization. Supportive of the latter hypothesis is that GC B cells have been found to persist for several weeks following immunization (27). High affinity B cell clones are selected to become long-lived PCs that migrate to the bone marrow (32). Vaccine platforms that are powerful at generating antibody responses have shown superior GC formation capacities (23, 33, 34). With the clear GC activation found at 2 weeks following boost immunization, additional affinity maturation and subsequent differentiation of long-lived PCs likely continued for some time thereafter. Since affinity maturation occurs before the differentiation of PCs, it is possible that the PCs seeded early into the bone marrow were replaced by higher affinity PCs derived from the GC reaction induced by the boost immunization. This would explain the increase in antibody avidity found here.

To improve the efficacy of vaccines, there has been much focus on identifying immunological events, including biomarkers, which lead to the production of high titer protective antibodies. Tfh cells and their circulating counterparts have emerged as key players for dictating the antibody responses (35). As mentioned previously, studies have shown that CXCR3+ cTfh cells correlate with high-avidity antibodies against influenza after vaccination in humans (17, 18). It has also been shown that cTfh cell levels can predict the seroconversion in humans to influenza vaccination (36). In rhesus macaques, CXCR3+ Tfh levels in LNs were shown to correlate with avidity, longevity, and neutralization capacity of HIV vaccine-induced antibodies (20). In line with these observations, we found that the mRNA/LNP vaccine platform specifically induced an increase in CXCR3+ cTfh cells, which correlated with increased IgG avidity. This could indicate that CXCR3+ cTfh cells are important for selecting and expanding B cells of high affinity. Whether inducing cTfh cells toward the CXCR3+ phenotype is caused by the vaccine platform or the HA antigen remains to be elucidated. To this end, a type I IFN-mediated Th1-polarization after mRNA vaccine administration has been described and shown to at least partly be caused by TLR7/8 ligation (37, 38).

*In vitro* studies have proposed that CXCR3+ cTfh cells preferentially provide help to memory B cells compared to naïve B cells (14). However, CXCR3+ and CXCR3− Tfh cells sorted from rhesus LNs showed that there was no difference in their B cell help capacity (20). Since the vaccinated animals in our study showed a rapid induction of memory B cells, plasmablasts, and PCs, there was clearly an efficient priming of naïve B cells despite being naïve to influenza.

Furthermore, it has been proposed that the main function of CXCR3+ cTfh cells is to select memory B cells of high affinity, leading to rapid expansion of this population upon new antigen encounter (17). With regards to influenza, where the circulating strain changes every year, the ability to select for high-affinity B cell clones against the circulating strain is critical. Recent studies have shown that the cTfh cells that increase in blood after influenza vaccination represent memory Tfh cells that have been reactivated upon antigen re-exposure (19). cTfh cells can home to the LNs and differentiate into GC Tfh cells to facilitate the GC response (39, 40). Induction of vaccine-specific cTfh cells is, therefore, a central mechanism in vaccine-mediated protection, since these cells facilitate quick re-stimulation of memory B cells in the GC reaction. We found H10-specific cells within the CXCR3+ cTfh cell population. As a considerable proportion of ICOS and PD-1 expression was lost during the overnight stimulation, the number of H10-specific CXCR3+ cTfh cells may be underestimated.

Since CXCR3+ Tfh1 cells have been shown to be inferior to CXCR3− Tfh2/17 cells at providing help to naïve B cells, it was suggested that an influenza vaccine inducing Tfh2/17 cells rather than Tfh1 cells would be preferable (14, 41). However, passive transfer of antibody clones against HA in mice showed that only Th1-polarized IgG2a, and not Th2-polarized IgG1, conferred protection against lethal challenge, despite that the antibodies had the same ability to bind the antigen (35, 42, 43). This was proposed to be due to the different Fc regions of the antibodies and indicates that antibodies generated by help from cTfh cells of the Th1 subtype may be critical for the induction of protection against influenza.

In summary, we show here that an influenza mRNA/LNP vaccine induces robust GC and B cell responses, including PCs seeding into the bone marrow. Antibody avidity increases over time and is accompanied by cTfh cells of the CXCR3+ subtype. Collectively, this gives insights into the adaptive immune profile generated by mRNA/LNP vaccines and may indicate that this platform is particularly powerful for infections such as influenza that require a Th1-profile.

## Ethics Statement

Chinese rhesus macaques were housed in the Astrid Fagraeus laboratory at Karolinska Institutet according to guidelines of the Association for Assessment and Accreditation of Laboratory Animal Care, and all procedures were performed abiding to the provisions and general guidelines of the Swedish Animal Welfare Agency. This animal study was approved by the Local Ethical Committee on Animal Experiments.

## Author Contributions

GL, FL, KB, SJ, KH, LB, HS, GC, and KL designed research. GL, SO, FL, ET, AL, FH, KB, SJ, KH, LB, HS, GC, and KL performed experiments and contributed with vaccines. GL, SO, FL, ET, FH, GC, and KL analyzed data. GL, SO, FL, ET, KB, and KL wrote the paper. All authors reviewed the paper.

## Conflict of Interest Statement

The authors declare that no conflict of interest exists. The authors KB, HS, KH, LB, HS, and GC are employees of Moderna Therapeutics.

## Supplementary Material

Table S1 List of antibodies used for flow cytometry.

Table S2 List of antibodies used for flow cytometry.

## Abbreviations

ASCs, antibody-secreting cells; cTfh, circulating T follicular helper; GCs, germinal centers; GLA, glucopyranosyl lipid adjuvant; HA, hemagglutinin; ID, intradermal; IM, intramuscular; LNP, lipid nanoparticle; LN, lymph node; PC, plasma cell; PD-1, programmed death receptor 1; Th, T helper; Tfh, T follicular helper.

## References

[1] Pardi N Hogan MJ Pelc RS Muramatsu H Andersen H DeMaso CR Zika virus protection by a single low-dose nucleoside-modified mRNA vaccination. Nature (2017) 543:248–51. 10.1038/nature21428 28151488 PMC5344708

[2] Richner JM Himansu S Dowd KA Butler SL Salazar V Fox JM Modified mRNA vaccines protect against Zika virus infection. Cell (2017) 168:1114–25.e10. 10.1016/j.cell.2017.02.017 28222903 PMC5388441

[3] Ulmer JB Geall AJ. Recent innovations in mRNA vaccines. Curr Opin Immunol (2016) 41:18–22. 10.1016/j.coi.2016.05.008 27240054

[4] Petsch B Schnee M Vogel AB Lange E Hoffmann B Voss D Protective efficacy of in vitro synthesized, specific mRNA vaccines against influenza A virus infection. Nat Biotechnol (2012) 30:1210–6. 10.1038/nbt.2436 23159882

[5] Bahl K Senn JJ Yuzhakov O Bulychev A Brito LA Hassett KJ Preclinical and clinical demonstration of immunogenicity by mRNA vaccines against H10N8 and H7N9 influenza viruses. Mol Ther (2017) 25:1316–27. 10.1016/j.ymthe.2017.03.035 28457665 PMC5475249

[6] Sahin U Kariko K Tureci O mRNA-based therapeutics – developing a new class of drugs. Nat Rev Drug Discov (2014) 13:759–80. 10.1038/nrd4278 25233993

[7] Coelho T Adams D Silva A Lozeron P Hawkins PN Mant T Safety and efficacy of RNAi therapy for transthyretin amyloidosis. N Engl J Med (2013) 369:819–29. 10.1056/NEJMoa1208760 23984729

[8] Plotkin SA. Correlates of protection induced by vaccination. Clin Vaccine Immunol (2010) 17:1055–65. 10.1128/CVI.00131-10 20463105 PMC2897268

[9] Victora GD Nussenzweig MC. Germinal centers. Annu Rev Immunol (2012) 30:429–57. 10.1146/annurev-immunol-020711-075032 22224772

[10] Nutt SL Hodgkin PD Tarlinton DM Corcoran LM. The generation of antibody-secreting plasma cells. Nat Rev Immunol (2015) 15:160–71. 10.1038/nri3795 25698678

[11] Crotty S Follicular helper CD4 T cells (TFH). Annu Rev Immunol (2011) 29:621–63. 10.1146/annurev-immunol-031210-101400 21314428

[12] Suan D Sundling C Brink R. Plasma cell and memory B cell differentiation from the germinal center. Curr Opin Immunol (2017) 45:97–102. 10.1016/j.coi.2017.03.006 28319733

[13] Morita R Schmitt N Bentebibel SE Ranganathan R Bourdery L Zurawski G Human blood CXCR5(+)CD4(+) T cells are counterparts of T follicular cells and contain specific subsets that differentially support antibody secretion. Immunity (2011) 34:108–21. 10.1016/j.immuni.2010.12.012 21215658 PMC3046815

[14] Schmitt N Bentebibel SE Ueno H. Phenotype and functions of memory Tfh cells in human blood. Trends Immunol (2014) 35:436–42. 10.1016/j.it.2014.06.002 24998903 PMC4152409

[15] Heit A Schmitz F Gerdts S Flach B Moore MS Perkins JA Vaccination establishes clonal relatives of germinal center T cells in the blood of humans. J Exp Med (2017) 214(7):2139–52. 10.1084/jem.20161794 28637884 PMC5502430

[16] Geginat J Paroni M Maglie S Alfen JS Kastirr I Gruarin P Plasticity of human CD4 T cell subsets. Front Immunol (2014) 5:630. 10.3389/fimmu.2014.00630 25566245 PMC4267263

[17] Bentebibel SE Khurana S Schmitt N Kurup P Mueller C Obermoser G ICOS(+)PD-1(+)CXCR3(+) T follicular helper cells contribute to the generation of high-avidity antibodies following influenza vaccination. Sci Rep (2016) 6:26494. 10.1038/srep26494 27231124 PMC4882544

[18] Bentebibel SE Lopez S Obermoser G Schmitt N Mueller C Harrod C Induction of ICOS+CXCR3+CXCR5+ TH cells correlates with antibody responses to influenza vaccination. Sci Transl Med (2013) 5:176ra132. 10.1126/scitranslmed.3005191 23486778 PMC3621097

[19] Herati RS Muselman A Vella L Bengsch B Parkhouse K Del Alcazar D Successive annual influenza vaccination induces a recurrent oligoclonotypic memory response in circulating T follicular helper cells. Sci Immunol (2017) 2:eaag2152. 10.1126/sciimmunol.aag2152 28620653 PMC5469419

[20] Iyer SS Gangadhara S Victor B Gomez R Basu R Hong JJ Codelivery of envelope protein in alum with MVA vaccine induces CXCR3-biased CXCR5+ and CXCR5- CD4 T cell responses in rhesus macaques. J Immunol (2015) 195:994–1005. 10.4049/jimmunol.1500083 26116502 PMC4506863

[21] Richner JM Himansu S Dowd KA Butler SL Salazar V Fox JM Modified mRNA vaccines protect against Zika virus infection. Cell (2017) 169(1):176 10.1016/j.cell.2017.03.016 28340344

[22] Spangberg M Martinez P Fredlund H Karlsson Hedestam GB Sundling C. A simple and safe technique for longitudinal bone marrow aspiration in cynomolgus and rhesus macaques. J Immunol Methods (2014) 408:137–41. 10.1016/j.jim.2014.05.004 24846526

[23] Liang F Lindgren G Sandgren KJ Thompson EA Francica JR Seubert A Vaccine priming is restricted to draining lymph nodes and controlled by adjuvant-mediated antigen uptake. Sci Trans Med (2017) 9:eaal2094. 10.1126/scitranslmed.aal2094 28592561

[24] Sundling C Martinez P Soldemo M Spångberg M Bengtsson KL Stertman L Immunization of macaques with soluble HIV type 1 and influenza virus envelope glycoproteins results in a similarly rapid contraction of peripheral B-cell responses after boosting. J Infect Dis (2013) 207:426–31. 10.1093/infdis/jis696 23162135

[25] Trombetta CM Perini D Mather S Temperton N Montomoli E. Overview of serological techniques for influenza vaccine evaluation: past, present and future. Vaccines (Basel) (2014) 2:707–34. 10.3390/vaccines2040707 26344888 PMC4494249

[26] Lamprecht M Sabatini D Carpenter A CellProfiler™: free, versatile software for automated biological image analysis. Biotechniques (2007) 42:71–5. 10.2144/000112257 17269487

[27] Havenar-Daughton C Carnathan DG Torrents de la Peña A Pauthner M Briney B Reiss SM Direct probing of germinal center responses reveals immunological features and bottlenecks for neutralizing antibody responses to HIV Env Trimer. Cell Rep (2016) 17:2195–209. 10.1016/j.celrep.2016.10.085 27880897 PMC5142765

[28] Havenar-Daughton C Lindqvist M Heit A Wu JE Reiss SM Kendric K CXCL13 is a plasma biomarker of germinal center activity. Proc Natl Acad Sci U S A (2016) 113:2702–7. 10.1073/pnas.1520112113 26908875 PMC4790995

[29] Hekele A Bertholet S Archer J Gibson DG Palladino G Brito LA Rapidly produced SAM((R)) vaccine against H7N9 influenza is immunogenic in mice. Emerg Microbes Infect (2013) 2:e52 10.1038/emi.2013.54 26038486 PMC3821287

[30] Osterholm MT Kelley NS Sommer A Belongia EA. Efficacy and effectiveness of influenza vaccines: a systematic review and meta-analysis. Lancet Infect Dis (2012) 12:36–44. 10.1016/S1473-3099(11)70295-X 22032844

[31] Michiels B Govaerts F Remmen R Vermeire E Coenen S. A systematic review of the evidence on the effectiveness and risks of inactivated influenza vaccines in different target groups. Vaccine (2011) 29:9159–70. 10.1016/j.vaccine.2011.08.008 21840359

[32] Weisel FJ Zuccarino-Catania GV Chikina M Shlomchik MJ. A temporal switch in the germinal center determines differential output of memory B and plasma cells. Immunity (2016) 44:116–30. 10.1016/j.immuni.2015.12.004 26795247 PMC4724390

[33] Ma Y Ross AC. Toll-like receptor 3 ligand and retinoic acid enhance germinal center formation and increase the tetanus toxoid vaccine response. Clin Vaccine Immunol (2009) 16:1476–84. 10.1128/CVI.00282-09 19692622 PMC2756847

[34] Martinez-Murillo P Tran K Guenaga J Lindgren G Àdori M Feng Y Particulate array of well-ordered HIV clade C Env trimers elicits neutralizing antibodies that display a unique V2 cap approach. Immunity (2017) 46(804–817):e807. 10.1016/j.immuni.2017.04.021 28514687 PMC5528178

[35] Michelle DLH Linterman A. Can follicular helper T cells be targeted to improve vaccine efficacy? F1000Res (2016). 10.12688/f1000research.7388.1 26989476 PMC4784016

[36] Pilkinton MA Nicholas KJ Warren CM Smith RM Yoder SM Talbot HK Greater activation of peripheral T follicular helper cells following high dose influenza vaccine in older adults forecasts seroconversion. Vaccine (2017) 35:329–36. 10.1016/j.vaccine.2016.11.059 27919633 PMC5191956

[37] Kranz LM Diken M Haas H Kreiter S Loquai C Reuter KC Systemic RNA delivery to dendritic cells exploits antiviral defence for cancer immunotherapy. Nature (2016) 534:396–401. 10.1038/nature18300 27281205

[38] Zhang X Casartelli N Lemoine S Mozeleski B Azria E Le Ray C Plasmacytoid dendritic cells engagement by influenza vaccine as a surrogate strategy for driving T-helper type 1 responses in human neonatal settings. J Infect Dis (2014) 210:424–34. 10.1093/infdis/jiu103 24558121

[39] He J Tsai LM Leong YA Hu X Ma CS Chevalier N Circulating precursor CCR7(lo)PD-1(hi) CXCR5(+) CD4(+) T cells indicate Tfh cell activity and promote antibody responses upon antigen reexposure. Immunity (2013) 39:770–81. 10.1016/j.immuni.2013.09.007 24138884

[40] Sage PT Alvarez D Godec J von Andrian UH Sharpe AH. Circulating T follicular regulatory and helper cells have memory-like properties. J Clin Invest (2014) 124:5191–204. 10.1172/JCI76861 25347469 PMC4348955

[41] Locci M Havenar-Daughton C Landais E Wu J Kroenke MA Arlehamn CL Human circulating PD-1+CXCR3-CXCR5+ memory Tfh cells are highly functional and correlate with broadly neutralizing HIV antibody responses. Immunity (2013) 39:758–69. 10.1016/j.immuni.2013.08.031 24035365 PMC3996844

[42] DiLillo DJ Tan GS Palese P Ravetch JV Broadly neutralizing hemagglutinin stalk-specific antibodies require FcgammaR interactions for protection against influenza virus in vivo. Nat Med (2014) 20:143–51. 10.1038/nm.3443 24412922 PMC3966466

[43] Snapper CM Paul WE. Interferon-gamma and B cell stimulatory factor-1 reciprocally regulate Ig isotype production. Science (1987) 236:944–7. 10.1126/science.3107127 3107127

