# Corrigendum: Induction of Robust B Cell Responses After Influenza mRNA Vaccination Is Accompanied by Circulating Hemagglutinin-Specific ICOS+ PD-1+ CXCR3+ T Follicular Helper Cells

**DOI:** 10.3389/fimmu.2019.00614

**Published:** 2019-04-02

**Authors:** Gustaf Lindgren, Sebastian Ols, Frank Liang, Elizabeth A. Thompson, Ang Lin, Fredrika Hellgren, Kapil Bahl, Shinu John, Olga Yuzhakov, Kimberly J. Hassett, Luis A. Brito, Hugh Salter, Giuseppe Ciaramella, Karin Loré

**Affiliations:** ^1^Department of Medicine Solna, Immunology and Allergy Unit, Karolinska Institutet, Stockholm, Sweden; ^2^Center for Molecular Medicine, Karolinska Institutet, Stockholm, Sweden; ^3^Valera LLC, Cambridge, MA, United States; ^4^Moderna Therapeutics, Cambridge, MA, United States; ^5^Department of Clinical Neuroscience, Karolinska Institutet, Stockholm, Sweden

**Keywords:** mRNA vaccine, adaptive immune responses, non-human primates, influenza, T follicular helper cells, germinal centers

In the original article, Liang et al. (44) was not cited in the article. The citation has now been inserted in the **Results, mRNA vaccine encoding H10 induces protective levels of antibodies**, paragraph two and should read:

“All animals induced neutralizing antibody titers against HA above the accepted level of protection for seasonal influenza vaccination, as measured by hemagglutination inhibition assay (HAI) (Figure 1C) as we have reported earlier (25, 44). Although some of the animals in the ID group already showed titers at the protective level after the prime immunization, all groups had titers that exceeded this level following boost. The antibody levels persisted above this level for the remainder of the study. The titers were significantly higher in the ID group compared to the IM groups for up to 2 weeks following boost, but were similar thereafter. The GLA group did not show higher HAI titers compared to the other groups, thus indicating that the mRNA/LNP formulation itself was sufficiently immunogenic. The third immunization in the GLA group resulted in a transient increase in HAI titers, which returned to similar levels as the other groups 5 weeks later.”

The authors apologize for this error and state that this does not change the scientific conclusions of the article in any way.
