# Supplementary material for: Induction of Robust B Cell Responses after Influenza mRNA Vaccination Is Accompanied by Circulating Hemagglutinin-Specific ICOS+ PD-1+ CXCR3+ T Follicular Helper Cells
Source: Front Immunol. 2017 Nov 13;8:1539. doi: 10.3389/fimmu.2017.01539 (PMC5693886; doi:10.3389/fimmu.2017.01539)
Supplement: Table S1 — List of antibodies used for flow cytometry. [file Table_1.docx]

Supplementary Table 1.

| **Marker** | **Clone** | **Company** |
| --- | --- | --- |
| CD3 | SP34-2 | BD |
| CD4 | S3.5 | ThermoFisher Scientific |
| CD28 | CD28.2 | Beckman Coulter |
| CD95 | DX2 | BD |
| CXCR3 | G025H7 | Biolegend |
| CD14 | M5E2 | Biolegend |
| CXCR5 | MU5UBEE | eBioscience |
| PD-1 | EH12.2H7 | Biolegend |
| ICOS | C398.4A | Biolegend |
| IFNy | 4S.B3 | BD |
|  |  |  |
|  |  |  |
|  |  |  |
|  |  |  |
|  |  |  |
|  |  |  |
|  |  |  |
|  |  |  |
|  |  |  |
|  |  |  |
|  |  |  |
|  |  |  |
|  |  |  |
|  |  |  |
