# Supplementary material for: Induction of Robust B Cell Responses after Influenza mRNA Vaccination Is Accompanied by Circulating Hemagglutinin-Specific ICOS+ PD-1+ CXCR3+ T Follicular Helper Cells
Source: Front Immunol. 2017 Nov 13;8:1539. doi: 10.3389/fimmu.2017.01539 (PMC5693886; doi:10.3389/fimmu.2017.01539)
Supplement: Table S2 — List of antibodies used for flow cytometry. [file Table_2.docx]

Supplementary Table 2.

| **Marker** | **Clone** | **Company** |
| --- | --- | --- |
| CD95 | DX2 | BD |
| IgM | G20-127 | BD |
| CD20 | 2H7 | Biolegend |
| CD3 | SP34-2 | BD |
| HLA-DR | L243 | Biolegend |
| Bcl6 | K112-91 | BD |
| Ki67 | B56 | BD |
|  |  |  |
|  |  |  |
|  |  |  |
|  |  |  |
|  |  |  |
|  |  |  |
|  |  |  |
|  |  |  |
|  |  |  |
|  |  |  |
|  |  |  |
|  |  |  |
|  |  |  |
|  |  |  |
|  |  |  |
|  |  |  |
|  |  |  |
